# Lectures on the Diagnosis and Treatment of Diseases of the Lungs

**Published:** 1840-09-12

**Authors:** W. W. Gerhard


MEDICAL EXAMINER.

DEVOTED TO MEDICINE, SURGERY, AND THE COLLATERAL SCIENCES.

No. 37.] PHILADELPHIA, SATURDAY, SEPTEMBER 12, 1840. [Vol. III.

### LECTURES ON THE DIAGNOSIS AND TREATMENT OF DISEASES OF THE LUNGS.

BY W. W. GERHARD, M. D.

### LECTURE X.

#### DILATATION OF THE BRONCHIAL TUBES.

There are two lesions of the bronchi, arising from long-continued bronchitis, which differ very widely, however, in their physical condition, viz. dilatation, and contraction of the bronchial tubes. The former of these lesions is by far the more important, and also the more frequent; and it prevails in proportion to the number of cases of long-continued chronic bronchitis, with abundant secretions. Acute bronchitis will occasionally produce the same dilatation, provided the mucus be copious, and be expectorated with difficulty after violent efforts of coughing; hence it is not infrequent in pertussis, which is about the only disease of children that gives rise to this lesion.

As dilatation of the bronchial tubes is a mere lesion, which is produced by diseased action, but is in itself of little importance, it must necessarily require less attention than the diseases of the lungs, properly so called: nevertheless, it may be readily confounded with these affections,—and even if it were not liable to this chance of error, there would still remain sufficient reason for studying the lesion. Lesions of this kind should never be confounded with the diseases which give rise to them; but they offer interesting points of relation, and require therefore some attention, in order to recognise them, and to discover the best means of obviating the mischievous effect which necessarily arises from their existence.

Dilatation of the bronchi assumes several different forms: the most frequent is an uniform enlargement of several bronchi of a lobe which, after branching off from the principal trunks, remain nearly of their original size, or even enlarge as they approach the surface of the lung. This variety results very frequently from whooping cough, and the spasmodic bronchitis which resembles it most nearly. The mucous membrane, at the same time, is thickened, and loses its transparency.

The other varieties, which are less common, are merely partial dilatations in the course of a bronchial tube. There may be only one single enlargement, or several successive dilatations of a large bronchus, which afterwards recovers nearly its natural size. The enlarged portions are thus distinct cavities, and physically speaking, present nearly the same peculiarities as the cavities which arise from the softening of tuberculous matter. There is therefore necessarily cavernous respiration and pectoriloquy, and the condensed pulmonary tissue which surrounds the enlargement may cause a decided dulness on percussion. The condensation of the tissue apparently arises from chronic inflammation, which causes a deposit of new matter in the pulmonary substance. The precise nature of this substance is not ascertained; but it is probably albuminous, like similar deposits in other parts of the body. In the variety in which the bronchial tubes are generally dilated, there is rarely cavernous respiration; for the air, in diffusing itself through the lung, does not, of course, present the sharp, clear reverberation, which is essential to the formation of cavernous respiration. Hence there is very little difference between the respiration in this variety of dilatation, and that heard in the second stage of pneumonia, when the hepatization occurs around the larger tubes. But in dilatation of the bronchi, the bronchial rhonchi, as the mucous and subcrepitant, are much more frequent than in pneumonia; and the permanency of the signs in the former alteration, and their rapid changes in the latter, will prevent all danger of confounding the two lesions together. The diagnosis between dilatation of the tubes and phthisis is much more difficult, as it depends not upon the physical signs, which differ but little in the two cases, but upon the progress of the general symptoms. If the symptoms be those of chronic bronchitis, that is, are attended with severe cough, and but slight emaciation, the disease is probably chronic bronchitis; but if the fever and emaciation be much more decided, the probabilities are of course greatly in favour of phthisis. Practically speaking, the chances of error are very slight; for those cases of chronic bronchitis, in which the dilatation of the tubes is sufficiently great to simulate a tuberculous cavity, are almost always connected with very chronic bronchitis, in which the signs of a general thickening and inflammation of the mucous membrane are very evident, and totally unlike those of a tuberculous disease. There is, however, another variety, in which it is impossible to discriminate accurately between these two affections, for the tuberculous disease then coincides with the dilatation of the tubes. In this case the dilated tubes either pass through the masses of tubercle which are deposited at the summit of the lungs, or they terminate as soon as they reach these masses. If the tubercles have advanced to the period of softening, the cavities which are thus produced often communicate directly with the enlarged bronchi, and form, as it were, a continuous tube. When the dilatation is connected with a cavity, it is often preceded by a deposit of tuberculous matter in the bronchus, which is in this way gradually enlarged, and remains dilated after the softening of its contents.

There is, of course, no peculiar treatment for the dilatation of the bronchi; it is strictly a lesion, not a disease, and being placed beyond the reach of the mechanical means of treatment which are adapted to remove an external alteration, it necessarily must remain with the patient. The object of the physician is to remove, as far as possible, the protracted bronchitis which generally produces the dilatation. The lesion then ceases to give rise to much mischief, and even a partial cure may take place.

### EMPHYSEMA OF THE LUNGS.

This is an alteration which is closely analogous with dilatation of the tubes. In fact, it is the same disease attacking a different part of the structure,—that is, the terminating vesicles of the lungs. In their normal state these cavities are very minute, but may still be discovered by a good eye; but when diseased their size may increase much beyond their natural dimensions, and they then very frequently attain the bigness of a small pea, and in some cases are even much larger. The vesicles, as they enlarge, at the same time become thickened in their parietes, and press upon those adjoining, of which some are atrophied, and others appear to form a direct connection with the distended ones. It is in this way that the very large sacs, of the size of a pigeon's, or even a hen's egg, seem to originate, not from a single vesicle, but from the junction of a number of distinct ones, which have gradually broken into each other.

The tissue of the lung which is the seat of the emphysema, becomes pale, and crackles under the pressure of the fingers like a piece of dried lung,—the walls of the vesicles losing their elasticity, and becoming much more rigid. The size of the dilated part of the lung is necessarily increased; hence it presses upon the intercostal spaces, and can no longer be confined in its usual limits. As a necessary consequence of this increase, the walls of the chest are enlarged to an extent corresponding with the distended part of the lungs, and form a decided protuberance.

The quantity of blood contained in an emphysematous lung is rather less than natural in those portions of it which are the especial seat of the disease,—that is, the anterior margin of it,—but the posterior parts contain as much blood as usual, and sometimes become congested on account of the dyspnœa, which is a necessary attendant upon all severe cases of the disease. The congestion frequently passes into pneumonia, and cases which prove fatal, for the most part terminate in this way. The mucous membrane of the bronchial tubes is rarely perfectly healthy in emphysema, if it be of severe character. There are two forms of bronchitis which commonly complicate emphysema,—the chronic, and the acute. The former is a regular, and almost necessary complication; the latter is often absent during nearly the whole course of the disease, but it is more apt to occur in patients labouring under this disorder than in those who are in the enjoyment of perfect health,—and when it takes place as a complication, the distress of the patient is vastly greater than in simple cases of bronchial inflammation. The chronic bronchitis which so commonly attends emphysema, is nearly always the dry bronchitis, or as it is often termed, the dry catarrh. In this variety the membrane is permanently thickened to such a degree as to impede the passage of the air, and constantly react upon the disease itself. The bronchitis is then doubly connected with the emphysema, and may be regarded both as cause and effect; either of the disorders may occur first, and will be almost necessarily followed by the other. Chronic dry catarrh produces of itself sufficient dyspnœa to distend the air-cells, and favour the development of emphysema; while if the anatomical condition exists, either as the result of original structure, or some peculiar cause, the slightest obstruction to the freedom of the respiratory function may cause a severe attack of dyspnœa, and thickening of the bronchial membrane is then almost a necessary result.

*Signs.*—The physical signs of emphysema are extremely well-marked in severe cases; but, of course, there are many instances in which the alteration deviates so little from the normal standard as to render the signs of doubtful value. When there is much distension, the physical signs are all present, and may be referred to the three following heads:—1. Distension of the portion of the chest. 2. Clearness of sound on percussion. 3. Feebleness of respiratory murmur. These are the only regular or constant signs, but there are occasionally a number of others perceived. These are sibilant rhonchus, from the frequent complication of dry catarrh; it is then heard along the anterior margin of the lungs: and subcrepitant, or mucous rhonchus, at the posterior part of the lungs, when they are much congested, or the bronchial tubes are attacked with acute inflammation. There is another sign which is occasionally met with,—the dry subcrepitant rhonchus, which is nothing but the slight rustling sound produced by the bubbles of air either forcing themselves into the cellular tissue and forming little bags which rub against the pleura, or the dilated vesicles themselves, which are sometimes sufficiently rigid to give rise to some friction.

1. *Dilatation of the Chest.*—This is necessarily most evident in those portions of the thorax where the dilatation of the vesicles is greatest; that is, at the anterior margin of the lungs. The anterior plane of the thorax is rounded, and gradually assumes a convex shape, the most prominent portion of it being near the margin of the sternum; the form of the dilated portion is generally oval, the long diameter of the oval corresponding to the axis of the body; but as the extent of the altered portion of the lung is very variable, the form of the chest differs extremely. The dilatation is more evident in the intercostal spaces than at the level of the ribs, which are but slightly thrown out from the general plane of the body. There is at times a general distension of the chest; the shoulders are then elevated, and rounded, and the thorax approaches very nearly to the cylindrical form. This extreme distension takes place only in those who have been long subject to emphysema, especially those who have inherited a predisposition to the disease. In speaking of the dilatation of the chest in emphysema, you must remember that it is moderate, and never attains the degree which we find in large pleuritic effusion, or in pneumothorax.

2. *Resonance on percussion.*—The anatomical condition of the lungs in emphysema necessarily admits more air into the lung,—in fact, the tissue is permanently distended with air,—and if percussion be made over the part, the sound is of course clearer than in a lung which is perfectly in the normal condition. This clearness is extremely great in thin persons who are affected with emphysema; if the patient be corpulent, and sufficiently advanced in life for the elasticity of the chest to be diminished, a moderate degree of emphysema does not render the percussion very sonorous. The clearness of sound is of course greatest at the spot where the dilatation is most perceptible; and when the chest is generally dilated, the percussion retains its character of great clearness throughout. The resonance in a few patients is sufficiently great to resemble that produced by pneumothorax, but it never has the tympanitic sound produced by the latter lesion.

3. *The feebleness of the respiratory murmur* is the third peculiarity of emphysema. The dilatation of the cells prevents a free circulation of air; they even remain permanently dilated when removed from the dead body. This immobility probably arises from the thickening of the walls of the vesicles, which always follows their permanent enlargement. The respiratory sound is not only enfeebled; but if the emphysema be extensive, it is apt to assume a peculiar rustling tone, which is probably in part produced by the vesicles themselves, and in part by their friction against the parietes of the chest.

The functional symptoms of emphysema are less characteristic than the physical signs, but are always sufficiently marked to increase the certainty of the diagnosis,—sometimes to indicate of themselves the character of the disease. One of them is much more constant than any other,—that is, the dyspnœa. The other symptoms depend in a great degree upon the complication of chronic or acute bronchitis, which so often attends the disease; hence they vary according to the intensity of this affection. They are cough, expectoration of thick, pearly sputa, which are small in quantity, or of a large amount of thin, glairy, and transparent matter, which occurs in the paroxysms of the dyspnœa, or during the complications of acute catarrh. There is no fever, or disturbance of other organs than the lungs or heart, which is necessarily connected with dyspnœa; when other affections occur, they may be set down as complications which may acquire additional severity from the pre-existence of the emphysema, but do not arise necessarily from it.

The dyspnœa is in part permanent, and in part comes on in paroxysms. The permanent dyspnœa is developed by any exercise which hurries the act of respiration, such as ascending a flight of stairs or a high hill, or indulging in any unwonted exercise. The subject of the disease then complains that he cannot take as much or as long-protracted exercise as other people; and this inability, if it be not accounted for by decided organic disease of the heart or lungs, is one of the best diagnostic characters of the disease. It is very regularly proportioned to the extent and severity of the emphysema, and in slight cases may escape notice. The dyspnœa which occurs in paroxysms is not frequent until the disease has become complicated with bronchitis, or as is still more frequent, with a disease of the heart. In the latter, a disturbance of the circulation is frequently produced by slight causes, and then the paroxysms of difficulty of respiration become extremely severe and intense, until the patient is partially relieved by a free expectoration of glairy mucus from the bronchial membrane, or until he remains for a considerable time in a condition of perfect repose. If the patient be extremely corpulent, the frequency of the paroxysms is of course proportionally increased, and they become more and more severe as the disease continues longer, for the dilatation in the majority of cases tends to increase,—and each successive attack, by distending the vesicles, may act as a new exciting cause of a further enlargement of them.

The diagnosis of severe cases of emphysema is readily enough made, for the physical signs are then pathognomic of the affection; but in slighter cases they are not always clearly enough developed to render the diagnosis quite certain. This is the case when there is little or no dilatation of the chest, but merely an increased resonance on percussion, and a diminished loudness of respiration. We are then obliged to resort to the diagnosis by way of exclusion; and if we find that no other disease which can account for the permanent dyspnœa exists, we should ascribe it to emphysema. When emphysema is complicated with another disease of the lungs, or with one of the heart which in itself is capable of producing a corresponding dyspnœa, it is difficult to ascertain the precise influence of the two affections. If the dyspnœa be excessive, emphysema alone is rarely capable of producing it; but if it be more moderate, the probable share of each affection is extremely difficult to ascertain.

The prognosis in this disease is favourable, so far as the chances of death are concerned,—for it is scarcely possible for a patient to die merely of emphysema. But, on the other hand, a complete recovery is scarcely possible, unless in very recent cases of the disease, when the distension of the air-cells has succeeded an acute disorder. In this case the disease tends gradually to recovery, although the restoration is rarely perfect; for the constant dilatation to which the vesicles are subjected prevents them from resuming their natural size.

The treatment of emphysema is in a great degree nugatory, so far as the removal of the lesion itself is concerned; but the paroxysms of dyspnœa may be checked, and the attacks of acute bronchitis relieved. The remedies most useful in checking the dyspnœa are sinapisms applied between the shoulders to the dorsal spinal vertebræ, and the use of lobelia in doses sufficient to excite slight nausea. If the tincture, which is the preferable form, be used, the dose should be twenty or thirty drops every two or three hours: some patients, however, will bear or even require a much larger dose, but for the greater number that just specified is sufficient. Opiates are also useful in some varieties of emphysema; they should be repeated often enough to quiet the cough; and in emphysema, as in common bronchitis, their effect is much enhanced by combining them with a nauseant. From a quarter to half a grain of opium will in general be found sufficient, if combined with the same or half the quantity of tartarized antimony. If ipecacuanha be used, of course the quantity should be larger. The remedies which are most serviceable for ordinary bronchitis, are of course equally applicable to that variety which complicates emphysema; it does not, therefore, require any specific direction for the treatment. It is to these cases that the physician is chiefly called; for in the large majority of cases the emphysema itself is not a sufficiently severe disease to attract much notice from the patient.

